# The use of purposeful sampling in a qualitative evidence synthesis: A worked example on sexual adjustment to a cancer trajectory

**DOI:** 10.1186/s12874-016-0114-6

**Published:** 2016-02-18

**Authors:** Charlotte Benoot, Karin Hannes, Johan Bilsen

**Affiliations:** Mental Health and Wellbeing Research Group (MENT), Vrije Universiteit Brussel, Laarbeeklaan 103, Brussels, 1050 Belgium; Centre for Sociological Research, Catholic University of Leuven, Parkstraat 45, Leuven, 3000 Belgium

**Keywords:** Qualitative evidence synthesis, Purposeful sampling, Sexual adjustment, Cancer treatment

## Abstract

**Background:**

An increasing number of qualitative evidence syntheses papers are found in health care literature. Many of these syntheses use a strictly exhaustive search strategy to collect articles, mirroring the standard template developed by major review organizations such as the Cochrane and Campbell Collaboration. The hegemonic idea behind it is that non-comprehensive samples in systematic reviews may introduce selection bias. However, exhaustive sampling in a qualitative evidence synthesis has been questioned, and a more purposeful way of sampling papers has been proposed as an alternative, although there is a lack of transparency on how these purposeful sampling strategies might be applied to a qualitative evidence synthesis. We discuss in our paper why and how we used purposeful sampling in a qualitative evidence synthesis about ‘sexual adjustment to a cancer trajectory’, by giving a worked example.

**Methods:**

We have chosen a mixed purposeful sampling, combining three different strategies that we considered the most consistent with our research purpose: intensity sampling, maximum variation sampling and confirming/disconfirming case sampling.

**Results:**

The concept of purposeful sampling on the meta-level could not readily been borrowed from the logic applied in basic research projects. It also demands a considerable amount of flexibility, and is labour-intensive, which goes against the argument of many authors that using purposeful sampling provides a pragmatic solution or a short cut for researchers, compared with exhaustive sampling.

Opportunities of purposeful sampling were the possible inclusion of new perspectives to the line-of-argument and the enhancement of the theoretical diversity of the papers being included, which could make the results more conceptually aligned with the synthesis purpose.

**Conclusions:**

This paper helps researchers to make decisions related to purposeful sampling in a more systematic and transparent way. Future research could confirm or disconfirm the hypothesis of conceptual enhancement by comparing the findings of a purposefully sampled qualitative evidence synthesis with those drawing on an exhaustive sample of the literature.

## Background

An increasing number of qualitative evidence synthesis papers are appearing in the health care literature [1, 2]. Qualitative evidence synthesis methods have the potential to generate answers to complex questions that provide us with novel and valuable insights for theory development and clinical practice, hereby moving beyond review questions only related to the effectiveness of interventions and causation [3, 4].

Over 20 different approaches to qualitative evidence synthesis have been developed [5]. Meta ethnography developed by Noblit and Hare (1988) is currently one of the most commonly used synthesis approaches [2, 6, 7]. Meta-ethnography enables a systematic and detailed understanding of how studies are related, through the comparison of findings within and across studies, ultimately providing an interpretation of the whole body of research [7]. It has known a considerable uptake in the field of healthcare [8, 9]. Furthermore, it has the capacity to generate hypotheses for future testing or comparison with trial outcomes [10]. In our review project, we opted for a meta-ethnographic approach to synthesize findings on the sexual adjustment of cancer patients and their partners across a number of qualitative studies. It was expected that this would allow us to generate a comprehensive model to understand patients and their partners’ sexual adaptation after cancer.

We noticed that many of the meta-ethnographies published adopt a linear approach to synthesizing the literature, mirroring the standard template developed by major review organizations such as the Cochrane and Campbell Collaboration. Consequently, in most meta- ethnographic synthesis projects, a strictly exhaustive search and information retrieval strategy is used to collect data and relevant studies are assessed for quality before being included in the synthesis. The idea to work with comprehensive samples of the literature is strongly influenced by the risk of bias discourse, suggesting that non-comprehensive samples may introduce a selection bias in systematic reviews, for example [11–13].

However, the usefulness of the review strategy promoted by organizations such as Cochrane and Campbell, and thus of exhaustive search techniques and sampling, has been questioned by a substantial proportion of members of the qualitative research community. It has been argued that exhaustive sampling is a highly rigorous and formalistic approach that risks to be too time consuming because the searches often retrieve very large data sets that are impractical to screen [14, 15]. Moreover, exhaustive sample risks to produce rather superficial synthesis findings, with a large number of studies that fail to go beyond the level of description [16].

Consequently, some authors are proposing a more purposeful way of sampling papers as an alternative for exhaustive sampling [17].

Purposeful sampling techniques for primary research have been well described by Patton (2002, p. 230) who has provided a definition of what purposeful sampling means [16].

“The logic and power of purposeful sampling lie in selecting information-rich cases for study in depth. Information-rich cases are those from which one can learn a great deal about issues of central importance to the purpose of the inquiry, thus the term purposeful sampling. Studying information-rich cases yields insights and in-depth understanding rather than empirical generalizations.”

Applied to the meta-level, purposeful sampling in a qualitative evidence synthesis has often been promoted as a solution for pragmatic constraints of time, resources, access to information and expertise [5, 15]. However, several review authors specializing in qualitative evidence synthesis have also provided a more theoretical background to the choice for purposeful sampling. One of the core arguments supporting a purposeful sampling approach is that it is not meant to be comprehensive in terms of screening all potentially relevant papers, mainly because the interest of the authors is not in seeking a single ‘correct’ answer, but rather in examining the complexity of different conceptualizations. It follows that these types of reviews require variation to enable new conceptual understandings to be generated [11, 17, 18]. Booth (2011) further claims that authors of qualitative evidence syntheses are mainly concerned with ‘aiming to find sufficient cases to explore patterns and so are not necessarily attempting to be exhaustive in their searching’ [19]. To guarantee a sufficient level of conceptual richness, review directions may be divergent and iterative, rather than linear [20]. This thus contradicts the classic prospective approach of exhaustive searching [1].

Although several qualitative researchers have recommended purposeful sampling in the context of qualitative evidence synthesis, the published literature holds sparse discussion on how these strategies might be applied to a qualitative evidence synthesis [15]. Suri (2011) has made a worthwhile attempt to address this issue by examining the adaptability of the 16 purposeful sampling strategies in primary research described by Patton (2002) to the process of qualitative evidence synthesis (see Table 1).

**Table 1:** Purposeful sampling strategies by Patton (2002), adapted by Suri (2011)

Purposeful sampling strategy	Purpose (Patton, 2002)	Purpose in qualitative evidence synthesis (Suri, 2011)
Extreme of deviant case sampling	Learning from highly unusual manifestations of the phenomenon of interest	Focusing on how things should be or could be, rather than how things are Suitable for realist syntheses
Intensity sampling	Information-rich cases that manifest the phenomenon intensely, but not extremely, such as good students/ poor students, above average/below average.	To develop a comprehensive understanding of the phenomena that is been researched in the synthesis
Maximum variation sampling	identifying key dimensions of variations and then finding cases that vary from each other as much as possible.	To identify essential features and variable features of a phenomenon among varied contexts
	Identifies important patterns that cut across variations	To construct an holistic understanding of the phenomenon
Homogenous sampling	Picking a small, homogeneous sample. Reduces variation, simplifies analysis, facilitates group interviewing	To overcome the critique of “mixing apples and oranges”:i.e. to overcome the epistemological incommensurability of different qualitative methods
		To describe some particular subgroup in-depth
		Suitable for participatory syntheses
Typical case sampling	Illustrates or highlights what is typical, normal, average	To study how common themes recurring in the published literature might be related to the relative strengths and weaknesses of the typical methodologies or theories underpinning the typical studies
Critical case sampling	Permits logical generalization and maxi-mum application of information to other cases	To assist stakeholders in making informed decisions about the viability of a certain innovation
Snowball sampling	Seeking information from key informants about details of other information-rich cases in the field	To identify studies that are highly valued by different stakeholders
		To identify studies outside the academic mainstream
Criterion sampling	Selecting all cases that meet some predetermined criterion of importance	To construct a comprehensive understanding of all the studies that meet certain pre-determined criteria
Theoretical sampling	Selecting cases that represent important theoretical constructs about the phenomenon of interest	Research synthesis who employ constant comparative methods or grounded –theory approaches
Confirming sampling	Selecting cases that are additional examples that fit already emergent patterns; these cases	To advocate a particular stance for ethical, moral and/or political reasons
		Suitable for openly ideological synthesis
Disconfirming sampling	Selecting cases that do not fit. They are a source of rival interpretations as well as a way of placing boundaries around confirmed findings	To shake our complacent acceptance of popular myths and generalizations in a field
Stratified purposeful sampling	Sampling within samples where each stratum is fairly homogeneous	To examine variations in the manifestation of a phenomenon as any key factor associated with the phenomenon is varied. In a research synthesis, this factor may be contextual, methodological, or conceptual.
Opportunistic sampling	Adding cases to a sample to take advantage of unforeseen opportunities after fieldwork has begun	To be used in a research area which is at its exploratory stage or when the synthesis does not have an insider status in the relevant field of research
		Suitable to participatory syntheses where the synthesis purpose evolves in response to the changing needs of the participant co-synthesists
Purposeful random sampling	Adds credibility to sample when potential purposeful sample is larger than one can handle. Reduces judgment within a purposeful category	To locate most of the primary research reported on a topic and then randomly select a few reports from this pool for in-depth discussion
Sampling politically important cases	Selecting a politically sensitive site or unit of analysis	To gain attention of different stakeholders and the synthesis findings get used.
		Suitable for synthesis of hot topics, in which several stakeholders are interested
Convenience sampling	Involve selecting cases that are easy to access and inexpensive to study	Not a recommendable technique, because its neither purposeful, nor strategic
Combination or mixed purposeful sampling	To use a combination of two or more sampling strategies to select evidence that adequately addresses their purpose	To facilitate triangulation and flexibility in meeting the needs of multiple stakeholders

Despite this promising effort by Suri (2011) to theoretically present the different options of sampling for synthesis, researchers who claim to have used a purposeful sampling approach often fail to create a transparent audit trail on the review process. In addition, early pioneers such as Campbell and colleagues (2003) who explored purposeful sampling remain close to a positivist sampling strategy, opting for an arbitrary, random sampling technique to select a subset of papers to extract [21]. Noblit and Hare (1988), the initiators of the meta-ethnographic approach, introduce the idea of sampling purposefully without developing it further [7].

This indicates that there is a unilateral focus on exhaustive sampling methods, as well as a lack of transparency on how to effectively use and report on purposeful sampling techniques. Therefore, we discuss in this paper why and how we have used purposeful sampling in our qualitative evidence synthesis. The following issues will be addressed: (a) how purposeful sampling procedures have been integrated into our review procedure; (b) how this purposeful sampling has led to the development of a line-of-argument, and (c) what sort of challenges and opportunities we encountered in the instrumental outline of the procedure.

## Methods

We used Suri’s (2011) description of 16 possible purposeful sampling strategies for qualitative evidence synthesis as a starting point for deciding on which type of sampling strategy we would apply in our synthesis (see Table 1) [15]. Suri (2011) urges authors to carefully identify sampling strategies that are conceptually aligned with the synthesis purpose, that are credible, that sufficiently address the synthesis purpose, and that are feasible, ethical and efficient.

However, we found that Suri did not offer a ‘grab and go’ option that was the perfect match for building a theoretical model, which was the aim in our qualitative evidence synthesis about sexual adjustment after cancer. Little guidance is thus available for the practical implementation of theoretical sampling. Following the example of theoretical sampling guides in primary research, we choose to see theoretical sampling as an umbrella approach, i.e. a combination of different purposeful sampling techniques [22, 23].

We have therefore chosen a combination consisting of (a) intensity sampling at first, then a (b) maximum variation sampling and finally (c) disconfirming case sampling. This combination of sampling techniques was chosen as these aligned with the different steps of analysing towards a theoretical construct, and in accordance with Corbin and Strauss, who also connected specific sampling strategies to different types of analysing [24].

In what follows, we describe and discuss how these sampling procedures have been integrated into our review procedure. As well we describe why we used the specific sampling technique in alliance with a specific step in the analysis.

1. **Scoping review**

Initially, we compiled a database of potentially relevant articles based on a scoping review. Scoping is an exploratory and systematic way of mapping the literature available on a topic [17]. Scoping exercises are perceived as the ideal way of doing preparatory work for an exhaustive systematic review. In our case, we have used them for building an archive of data for our qualitative evidence synthesis.

We searched 4 major databases: Medline, Psychinfo, Cinahl and Dissertation Abstracts. A search string was developed for each database with the support of a specialized team. For each database we added a methodological filter to these search strings in order to extract qualitative research articles [25–27]. For example, the research string we used in Medline was ((interview* or qualitative or experience*) and (cancer and sexual*). Studies included had to be written in English and be carried out between 1994 and 2014, for pragmatic reasons.

The qualitative studies retrieved were qualitative studies matched against the following inclusion criteria.

A. Type of studies
  We considered all sorts of qualitative designs. Opinion pieces and editorials were excluded. The study reports should be qualitative in nature.
B. Phenomenon of interest
  Studies should (partially) focus on the relational aspects of sexuality, namely the sexual intimacy of patient and partner, in a context of a cancer diagnosis.
C. Type of participants

We included articles where the cancer patient and/or the partner was the unit of analysis.

**Fig. 1:**
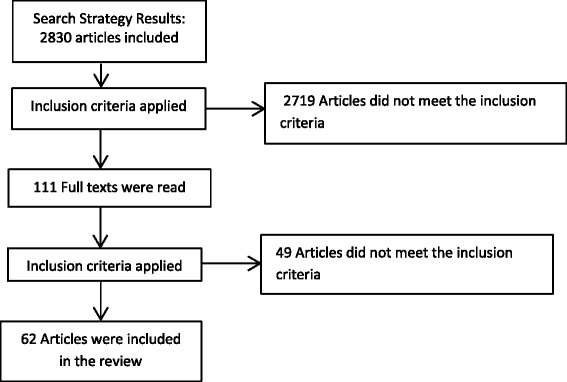
Flow chart of the scoping review

First one researcher (CB) applied the inclusion and exclusion criteria to the retrieved abstracts. A full text was requested for each of the relevant studies. These studies were further assessed by the same researcher, rechecking them against the same inclusion and exclusion criteria. As can be seen in Fig. 1, a total of 58 articles were included in our pool/archive of data.

The quality of the 58 studies was appraised using the CASP (Critical Appraisal Skills Program) tool, as this proved to be the most feasible instrument to appraise qualitative studies (Hannes, Lockwood, & Pearson, 2010). The appraisal of the quality of the research articles was not meant as an inclusion tool in scoping, but was used later on as a parameter for intensity sampling (see further).

**Fig. 2:**
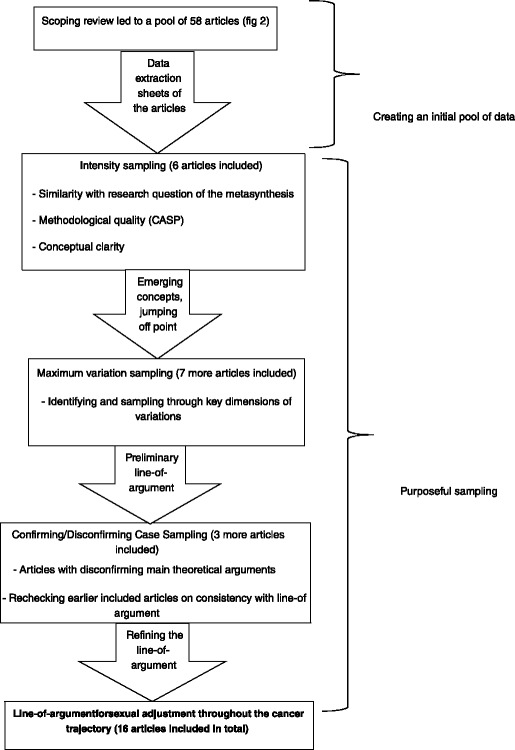
Overview figure of the purposeful sampling guidance

The pool of 58 data was used to initiate purposeful sampling –i.e. (a) intensity sampling, (b) maximum variation sampling, and (c) confirming/disconfirming case sampling (see Fig. 2).

In order to prepare for the purposeful sampling phase, we constructed a standardized extraction form for each of the 58 articles to highlight the specific characteristics identified, i.e. the data collection, method, research question/goal, sampling characteristics and main theoretical arguments. By summarizing the methodological and theoretical basis of the primary studies we could easily compare the differences between studies. This facilitated our choice in purposefully sampling papers. Table 2 shows an example of a descriptive data extraction sheet of one of the studies included.

**Table 2:** Example of descriptive data extraction sheet

	Walker (2012)
Data collection	Interviews together as a couple, unstructured interviews
Method	Grounded theory methodology
Research question/ goal	To present the struggles that these couples faced when trying to adapt sexually to the side effects of prostate cancer treatment
Sample characteristics (Age, sex)	18 heterosexual couples
	(m 47-83 years)
	(f 32 -82 years)
	Age patients: 65,4 y
	Age partners: 61 y
	Ethnicity: Euro-canadian or American heritage, 1 who was Afro-American
	Type of cancer treatment: Prostate cancer, all undergoing Adrogen Deprivation Therapy
Concepts	*an uncomfortable feeling about masturbation the avoidance of the topic of sexuality by the partners the more romantic husband ….*
Main theoretical arguments	*Whether couples choose to maintain sexual activity or cease engaging in sexual activity, they BOTH encounter a variety of struggles and for both choices, these struggles can be successfully overcome*

2. **Purposeful sampling**
  - Intensity sampling

“Intensity sampling in a research synthesis would involve selecting studies that are ‘excellent or rich examples of the phenomenon of interest, but not highly unusual cases [16]”.

The reason why we chose this sampling technique as the first technique is because we believed that the starting point of the literature synthesis would influence the further analysis, so it was important to choose rich examples of the phenomenon of interest, but not highly unusual cases.

The first task was to translate the theoretical definition of intensity sampling into some concrete inclusion factors. The first factor was the degree of overlap between the research question of the article and those of the qualitative evidence synthesis, because the content of the article had to parallel the intended content of our meta-ethnography closely. The second factor was the methodological quality of the paper, evaluated by means of the CASP. High-quality articles are usually more likely to provide rich, textual accounts to draw information from [28]. A third factor we assessed was the conceptual clarity of the article [29]. Conceptual clarity means the presence and clarity of concepts for translation, and is integral to a meta-ethnography which requires clear concepts as data.

We did this intensity sampling until a “jumping off point” was reached [30]. This point is reached when the concepts and categories emerging from the papers are saturated, meaning that no new concepts are derived from reading further articles. We retrieved this jumping off point after including 6 articles (see Fig. 2). From that point on, we wanted to deepen the concepts further by investigating the relation between the different concepts, by means of maximum variation sampling.

b. Maximum variation sampling

“A maximum variation sample is constructed by identifying key dimensions of variations and then finding cases that vary from each other as much as possible. This sampling yields: ‘(1) high-quality, detailed descriptions of each case, which are useful for documenting uniqueness, and (2) important shared patterns that cut across cases and derive their significance from having emerged out of heterogeneity [16].

Presuming that different study characteristics illuminate different aspects of a phenomenon, maximum variation sampling can be utilized to construct a holistic understanding of the phenomenon by synthesizing studies that differ in their study designs on several dimensions [15]. This type of sampling fits the stage of analysis as the aim is to uncover a many different key dimensions as possible.

The different concepts derived from the intensity sampling, defined the key dimensions that served as a basis for selecting additional papers. These papers vary from each other in these particular dimensions, e.g. theoretical underpinning of the articles (see further for an example of these key dimensions). Maximum variation sampling led us to the construction of a preliminary line-of-argument, after including 7 more articles (see Fig. 2) which was then further refined by using confirming/disconfirming case sampling.

c. disconfirming case sampling

“The disconfirming case sampling contains a selection of articles that do not fit [ the emerging patterns]. They are a source of rival interpretations as well as a way of placing boundaries around confirmed findings” [15].

Disconfirming case sampling fits this stage of analysis, as we want to verify and deepen the preliminary line of argument.

We selected new articles based on deviant theoretical assumptions. Disconfirming articles were thus also selected through the data extraction sheets of each paper, namely by reading through the main theoretical aspects of the studies. Papers that featured theories and concepts opposing the ones we had already included in our preliminary line-of-argument were further considered for in-depth analysis. We included 3 more articles for this sampling technique, which makes the total number of included articles 16 (see Fig. 2).

We have now addressed how to potentially introduce purposeful sampling into a review project. However, it has been suggested that a purposeful sampling procedure is subject to a permanent dialogue with the analysis of the data [31, 32]. In what follows, we will discuss what sort of contribution purposeful sampling has made to our findings and the model we have developed, by means of a worked example.

## Results: Illustration of the purposeful sampling techniques using a worked example

In a meta-ethnography, a popular way of analysing data is the translation of the concepts or metaphors of one study into another, while preserving the structure of relationships between concepts within any given study [33]. We will thus show how we sampled different studies and how this influenced the translation exercise based on an example of three example concepts from three articles included in our review. Note that the decision to work with three concepts only was taken to increase the clarity of the procedures we describe in this paper, not to describe all the actual results and complete line-of-argument.

1. **First step: Arriving at a “jumping off point” through intensity sampling**

We will illustrate these decisions of intensity sampling by describing the inclusion of 3 articles [34–36] which - according to our parameters described above - have a great degree of overlap with the research goal, a high methodological quality and strong conceptual clarity.

On the articles that were included through intensity sampling, we performed a reciprocal translation of the concepts, which is the translation of one study’s findings into another, using metaphors and overarching concepts. [7] In what follows, we give a worked example of how we did this reciprocal translation for 3 concepts identified in the initial set of studies considered for the synthesis, as this is a necessary step towards the illustration of the subsequent sampling methodology. In order to be explicit about how the concepts compared to one another, we created a table into which we placed and compared the concepts of each paper (See Table 3). Each row of the table represents a key concept. In the left collumn, we labelled the rows with concepts that encompassed all the relevant concepts from each paper.

**Table 3:** Intensity sampling: Example of reciprocal translation of 3 concepts

Concepts	Walker (2011)	Gilbert (2010)	Juraskova (2003)
Sexual struggling	Having a sense of loss	Altered body image	Reduced vaginal lubrication
Exacerbation of struggling	Avoiding communication about the sexual changes	Sticking to a coital imperative	Receiving radiotherapy combined with external radiation and brachytherapy
Sexual adjustment	Accepting the decision to stop sexuality	Renegotiating the practices of sexual intimacy	Sexual adjustment and quality of life

The first concept we retrieved through intensity sampling is “sexual struggling”, encompassing the different ways of struggling with the sexual changes due to cancer. In Walker’s study (2011) it is formulated as having a sense of loss [35]. In the study of Gilbert (2013), this is formulated as patients having an altered body image [36]. In Juraskova’s study (2013) it is formulated as “reduced vaginal lubrification” [34].

Another overarching concept that we retrieved was “exacerbation of struggling”, encompassing strategies, situations, characteristics that were leading to an increasing struggling with the sexual changes. In Gilbert’s study (2013), this is formulated as “sticking to the coital imperative”, which means that intercourse is the most normal and natural form of heterosexuality, and condemns those who cannot perform as dysfunctional. In Walker’s study (2012), this is formulated as avoidance of communication about the sexual changes. In Juraskova (2003), exacerbation of struggling is the case when the patients are “ Receiving radiotherapy combined with external radiation and brachytherapy”.

A third overarching concept we found was the “sexual adjustment” to changes due to having cancer, encompassing the different ways of adaptation to sexual changes. Gilbert’s study (2010) describes that there is “a renegociation of the practices of sexual intimacy”, which means that the couple included sexual practices that had previously been marginalized in relation to sexual intercourse. Walker (2011) formulates this adjustment as “accepting the decision to stop sexuality”. Juraskove (2003) formulates it as “sexual adjustment and quality of life”.

The articles were sampled by the main author, but all articles included by intensity sampling were read and analysed by two authors (CB and MS). After a certain point which we call the “jumping off point”, we began to discover certain key dimensions of variation between the studies, which we explored further through maximum variation sampling. In the worked example that we explain here was the discovery that the studies varied on the scientific approach they took on, resulting in a different interpretation of the overarching concepts. To illustrate this: Gilbert (2010) used a social-constructionist lens to investigate sexual adjustment, Walker (2011) used a more psychological approach to investigate the subject, and Juraskova (2003) underscores more the biological aspects of sexual changes after cancer. Through the maximum variation sampling, we thus want to further explore how these different approaches lead to different interpretations of the phenomenon.

2. **Second step: Apply a maximum variation sampling strategy to construct a preliminary line of argument**

To explore the consequence of variation on the key dimension, we used maximum variation sampling to include studies that varied on the above cited dimension (i.e. scientific approach, socio-, psycho, or biological perspective). In this worked example, we show through the inclusion of three more papers [37–39] how we arrived – through comparison of the papers- at a preliminary line of argument.

The sampling was also done by one researcher, but the articles were read and analysed by 2 researchers. As a result of this maximum variation sampling and constant comparison between the papers, could develop relationships between the different concepts and constructing a preliminary line of argument (see Table 4).

**Table 4:** Maximum variation sampling

	Walker ( 2011) + *Hanly (2014)*	Gilbert (2010) + *Fergus (2002)*	Juraskova (2003) + *Hartman (2014)*
struggling	Having a sense of loss + *Anger, depression* **Grieving about sexual changes**	Altered body image + *Identity struggle* **Sexual changes as biographical disruption**	Reduced vaginal lubrication *+* *loss of libido* **Sexual dysfunctions**
Exacerbation of struggling	Avoiding communication about the sexual changes + *Minimization of side effects* **Denial as one of the grief stages**	Sticking to a coital imperative + *Flaunting sexual prowess despite erectile function* **Following hegemonic discourses of sexuality**	Receiving radiotherapy combined with external radiation and brachytherapy + *unpredictability of the side-effects* **Characteristics of the cancer treatment**
Sexual adjustment	Accepting the decision to stop sexuality + *Accepting sexual changes* **Acceptance of sexual changes**	Renegociating the practices of sexual intimacy + *Redefinition of what sexuality means* **Sexual rediscovery**	Sexual adjustment and quality of life + *Using Viagra leads to sex similar to before cancer* **Sexual recovery**
**Line of arguments**	**= Sexual adjustment as a grieving process**	**= Sexual adjustment as a cognitive restructuring process**	**= Sexual adjustment as a rehabilitation process**

Note 1: The discursive parts are the concepts coming from the included papers as a result of maximum variation sampling

Note 2: The bold parts are new findings resulting from maximum variation sampling

First, with regard to the concept of struggling, we found that articles who work with a psychological approach, describe the concept of struggling on an emotional level, analog with the stages of grief (anger, depression,..) while the sociological articles describe it more on a level of identity, analog with the theory of biographical disruption. Articles who have a more biological approach reduce the struggling on a level of sexual dysfunction.

Second, with regard to the concept of exacerbation of struggling, articles who work with a psychological approach again describe a stage of the grief theory, which is denial. Sociological oriented articles work with the adherence to hegemonic discourses, and biological oriented articles use certain characteristics of the cancer treatment as barriers towards adjustment.

Third, with regard to the concept of sexual adjustment, articles who are psychological oriented again use a stage of the grief theory to encompass this adjustment, which is acceptance. Sociological oriented article worked with a “rediscovery” of what sexuality is. The changes are thus not merely accepted, rather they are incorporated in a new definition of the self and sexuality. Biological oriented articles worked with “sexual recovery”, which –in contrast to the sociological oriented articles- means that there is no difference in what sexuality means , but a reuptake of sexual activity , similar to what it was before the cancer.

Our preliminary line of argument consisted of three different pathways the articles worked with. First, there are articles following the grief theory to describe the adjustment process In this case, sexual changes are depicted in terms of losses, and the adjustment occurs through the process of grief and mourning.

Second, there are articles following the “restructuring theory” during illness. Unlike the case of grief theory, where the patient and partner are working through some emotional stages, in the restructuring pathway patient and partner are more cognitively dealing with sexuality after cancer through the development of a new sexual paradigm. Flexibility is the central aspect of this adjustment.

Thirdly, there are articles following the pathway of sexual rehabilitation. This pathway is embedded in a more positivistic paradigm where the adaptation does not emphasize psychological changes or cognitive restructuring, but sexual changes as a bodily dysfunction that needs treatment and behavioural strategies.

3. **Refining the preliminary line of argument by means of disconfirming case sample.**

To test, refine, and deepening our preliminary line-of-argument, , we included 3 articles out of the pool of 58 articles that consist of a theory and concepts opposing the preliminary line-of argument. We will give an example with including 1 article (see Table 5).

**Table 5:** Disconfirming case sampling

	White (2014)	Navon (2003)
Main theoretical arguments	The women colluded with the medicalization of their bodies which helped their adjustment	Despite the deceptive nature of the strategies of this patients, they are considered to be beneficial and even essential. However, their effectiveness diminishes over time due to the increasing salience of their self-deceptive nature

In this phase of sampling, we worked together with a researcher who was not involved in the analysis process before (JB). This is because we wanted to have a fresh and “unambiguous view” of our line of argument. This researcher, together with the first researcher, read the articles and tested them against the line of argument.

In our preliminary line-of-argument, we assumed that the three pathways of adjustment all followed a linear pattern from the struggling towards the adjustment. However, Ramirez (2009) counter argues this linear approach by stating that patients could refine their definition of sexuality, but could also return to it at a certain moment [40]. These disconfirming findings led us to re-analyse the included articles, where we came eventually to the conclusion that the sexual adjustment as a cognitive restructuring process does not have a linear pattern with an endpoint, but rather makes on oscillating movement between following hegemonic definitions of sexuality, and challenging them.

4. **Challenges and opportunities**

In the process of conducting a qualitative evidence synthesis through purposeful sampling, we encountered several challenges. But this process also created a few opportunities that would not have occurred if we had used an exhaustive sampling and analysis strategy. In what follows, we discuss how we have bridged obstacles and maximized benefits in terms of the opportunities arising.

First, it proved to be difficult to define what exactly to look for, since the concept of e.g. an intensity sample on the meta-level could not readily been borrowed from the logic applied in basic research projects. In an original research project, as opposed to a qualitative evidence synthesis project, purposeful sampling can often easily be conducted, for example by using a brief questionnaire as a screening tool to search for participants with specific characteristics [41]. However, with research reports, this is more difficult in practice. We chose to search for literature by means of electronic databases with the use of search strings. Finding a specific search string to detect a specific information-rich research report which meets the sampling criteria would be difficult, because the search terms are usually based on population and setting characteristics as well as the topic of interest, rather than on conceptual or theoretically interesting leads.

Therefore we decided to conduct a scoping of the literature prior to applying a purposeful sampling technique. The scoping review was intended to create a pool/or archive of primary research reports that are easily accessible and can be used later as material for purposeful sampling. In fact, our purposeful sampling strategy did not start at the level of data-collection. It was initiated at the level of data extraction and analysis. The consequence of this decision was that the sampling procedure was rather labour-intensive as we had to perform a scoping review before the actual mixed purposeful sampling could start.

We illustrated through our worked example that using purposeful sampling techniques also has several advantages.

First of all, although some researchers argued that reducing the number of included articles by means of purposeful sampling could result in neglecting important data [18, 42], we showed throughout this worked example that the opposite can be true. With the use of this combination of three purposeful sampling techniques – intensity sampling, maximum variation sampling and confirming/disconfirming case sampling - we arrived at a line-of-argument.

Because of this emphasis on conceptual robustness instead of generalization of the data, we were more sensitive to “deviant data”, i.e. data that may not have been picked up when synthesizing information from an exhaustive sample of the literature, because review authors are generally more focused on detecting commonalities between articles. When using an exhaustive sampling technique, researchers will arrive at results that describe the “greatest common devisor” of all included papers.

Furthermore, deviant data that has been derived through maximum variation sampling and confirming/disconfirming case sampling may add new perspectives or a new space of understanding to the line-of-argument, while sampling randomly may run the risk of preventing enhanced insight and knowledge.

Moreover, the combination of sampling techniques – instead of a random sample or just one method of purposeful sampling- could enhance the quality and diversity of the papers being included, and could make the results more conceptually aligned with the synthesis purpose. This would further enhance the possible impact a qualitative evidence synthesis could have on informing healthcare practice [43].

Such an approach, however, demands a considerable amount of flexibility from review authors, mainly because inclusion criteria may change progressively during the process. This fact, together with the experience described above of doing a labour-intensive scope of the literature, goes against the argument of many authors [5] that using purposeful sampling provides a pragmatic solution or a short cut for reviewers who have limited time for searching and screening. However, we felt we did gain some time in the analytical process, since the number of articles from which data were extracted was modest in number. This strategy is therefore recommended for authors who are left with a high number of relevant articles after screening for inclusion.

However, the choice of using this particular combination of sampling techniques should also be motivated from a theoretical perspective. Authors who want to build a theoretical model out of the qualitative evidence synthesis could use this scheme of sampling methods, as it aligns well with the different stages of analysis, and is parallel to what Corbin and Strauss suggested for primary research [24].

## Conclusion

In this paper, we addressed two different needs:

Firstly, we met the need for a transparent worked example of how to apply purposeful sampling techniques to a qualitative evidence synthesis. We believe that this paper can help other researchers to make decisions related to purposeful sampling in a more systematic and transparent way.

Secondly, we gave evidence for the beneficial effects of using purposeful sampling techniques in a qualitative evidence synthesis. Although purposeful sampling is a time-consuming activity that requires a lot of resources and flexibility from the researchers, it creates potential to arrive at a rich conceptual model that can be useful for clinical practice. Future research could confirm or disconfirm the hypothesis of conceptual enhancement by comparing the findings of a purposefully sampled qualitative evidence synthesis with those drawing on an exhaustive sample of the literature.
